# Palm‐Sized Lab‐In‐A‐Magnetofluidic Tube Platform for Rapid and Sensitive Virus Detection

**DOI:** 10.1002/advs.202310066

**Published:** 2024-04-17

**Authors:** Ziyue Li, Shuo Zhang, Jiongyu Zhang, Lori Avery, David Banach, Hui Zhao, Changchun Liu

**Affiliations:** ^1^ Department of Biomedical Engineering University of Connecticut Health Center Farmington Connecticut 06030 USA; ^2^ Department of Biomedical Engineering University of Connecticut Storrs Connecticut 06269 USA; ^3^ Department of Pathology and Laboratory Medicine University of Connecticut Health Center Farmington Connecticut 06030 USA; ^4^ Department of Medicine Division of Infectious Diseases University of Connecticut Health Center Farmington Connecticut 06030 USA; ^5^ Department of Mechanical Engineering University of Nevada Las Vegas Nevada 89154 USA

**Keywords:** CRISPR‐based nucleic acid detection, HIV detection, magnetofluidic separation, microfluidic technology, SARS‐CoV‐2 detection

## Abstract

Simple, sensitive, and accurate molecular diagnostics are critical for preventing rapid spread of infection and initiating early treatment of diseases. However, current molecular detection methods typically rely on extensive nucleic acid sample preparation and expensive instrumentation. Here, a simple, fully integrated, lab‐in‐a‐magnetofluidic tube (LIAMT) platform is presented for “sample‐to‐result” molecular detection of virus. By leveraging magnetofluidic transport of micro/nano magnetic beads, the LIAMT device integrates viral lysis, nucleic acid extraction, isothermal amplification, and CRISPR detection within a single engineered microcentrifuge tube. To enable point‐of‐care molecular diagnostics, a palm‐sized processor is developed for magnetofluidic separation, nucleic acid amplification, and visual fluorescence detection. The LIAMT platform is applied to detect SARS‐CoV‐2 and HIV viruses, achieving a detection sensitivity of 73.4 and 63.9 copies µL^−1^, respectively. Its clinical utility is further demonstrated by detecting SARS‐CoV‐2 and HIV in clinical samples. This simple, affordable, and portable LIAMT platform holds promise for rapid and sensitive molecular diagnostics of infectious diseases at the point‐of‐care.

## Graphical abstract

A simple, fully integrated, lab‐in‐a‐magnetofluidic tube (LIAMT) platform is developed for “sample‐to‐result” molecular detection of virus. The LIAMT device integrates viral lysis, nucleic acid extraction, isothermal amplification, and CRISPR detection by coupling with a palm‐sized processor. The LIAMT platform is clinically validated to detect SARS‐CoV‐2 and HIV in clinical samples.

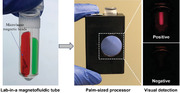

## 1 Introduction

Simple, rapid, and sensitive nucleic acid‐based molecular detection of virus is essential for monitoring and controlling the spread of infectious diseases, including COVID‐19 caused by the severe acute respiratory syndrome coronavirus 2 (SARS‐CoV‐2) and acquired immune deficiency syndrome caused by human immunodeficiency virus (HIV)^[^ ^1^, ^2^, ^3^, ^4^, ^5^ ^]^. To achieve highly sensitive and specific molecular detection in clinical samples, nucleic acid‐based diagnostics typically consist of three major steps: i) nucleic acid extraction, ii) enzymatic amplification, and iii) signal detection^[^ ^6^, ^7^, ^8^ ^]^. Polymerase chain reaction (PCR)/reverse transcription PCR (RT‐PCR) is considered the “gold standard” for nucleic acid amplification testing due to its high sensitivity and specificity^[^ ^9^, ^10^, ^11^ ^]^. However, current PCR/RT‐PCR assays require expensive instrumentation for precise thermal cycling, which limits the feasibility for low‐cost point‐of‐care diagnostic applications. Therefore, there is an unmet need to develop a simple, integrated, “sample‐to‐result” molecular diagnostic tool that can be used at the point‐of‐care, particularly in resource‐limited settings.

In recent decades, researchers have explored different strategies to develop simple, rapid, and affordable point‐of‐care diagnostic technologies for infectious disease detection^[^ ^12^, ^13^, ^14^, ^15^, ^16^ ^]^. Some studies have reported nucleic acid extraction‐free molecular diagnostic assays by combining simple heat‐treated sample preparation with nucleic acid amplification tests such as PCR and loop‐mediated isothermal amplification^[^ ^12^, ^13^, ^14^ ^]^. However, detecting low‐abundance target molecules in clinical samples without nucleic acid extraction and purification remains challenging. To meet stringent sensitivity requirements in clinical testing, solid phase extraction of nucleic acids has been integrated into microfluidic chips to develop fully integrated molecular diagnostic platforms, using extraction methods such as membrane‐based solid phase extraction^[^ ^15^, ^16^ ^]^ and magnetic bead‐based extraction^[^ ^17^, ^18^ ^]^. Among these methods, magnetic bead‐based solid phase extraction is widely used for integrated nucleic acid‐based molecular diagnostic platforms due to its speed, simplicity, and seamless integration with microfluidic technology^[^ ^17^, ^18^, ^19^, ^20^ ^]^. However, most extraction approaches depend on traditional PCR technology or single isothermal amplification assays, increasing the cost of instruments for precise temperature control or potentially causing false‐positive signals due to non‐specific amplification.

Recently, CRISPR technology has emerged as a powerful tool for nucleic acid‐based molecular detection due to its simplicity, robustness, and high specificity^[^ ^21^, ^22^, ^23^, ^24^, ^25^ ^]^. In particular, researchers have developed several highly sensitive and specific CRISPR‐based molecular diagnostic platforms, such as specific high‐sensitivity enzymatic reporter unlocking (SHERLOCK)^[^ ^21^ ^]^ and DNA endonuclease‐targeted CRISPR trans reporter (DETECTR)^[^ ^23^ ^]^, by combining CRISPR detection with isothermal amplification technologies (e.g., recombinase polymerase amplification [RPA]). However, most of these CRISPR assays are limited to detecting purified nucleic acid samples and lack a “sample‐to‐result” detection capacity. Typically, nucleic acid extraction and purification methods require bulky instruments (e.g., centrifuge machines) and multiple manual operations, which is not ideal for point‐of‐care diagnostic applications.

In this study, we describe the development of a simple, palm‐sized, lab‐in‐a‐magnetofluidic tube (LIAMT) platform for molecular detection of viruses. The LIAMT system is a self‐contained, all‐in‐one, microfluidic device that leverages the magnetofluidic transport of micro/nano magnetic beads, enabling “sample‐to‐result” nucleic acid testing. The device can perform viral lysis, nucleic acid extraction, reverse transcription RPA (RT‐RPA) and CRISPR‐based molecular detection. To enable simple point‐of‐care molecular diagnostics, we further developed a palm‐sized processor for magnetofluidic operation, isothermal amplification, and visual fluorescence detection. The detection signals can be visually observed by the naked eye or recorded by a smartphone, eliminating the need for an expensive optical instrument. We applied the LIAMT platform to detect both SARS‐CoV‐2 and HIV. To demonstrate its clinical utility, we further utilized the LIAMT platform to detect these viruses in clinical samples, thus demonstrating great promise for simple, sensitive, and affordable point‐of‐care diagnostics of infectious pathogens in resource‐limited settings or even at home.

## 2 Results and Discussion

### 2.1 Overview of the LIAMT Platform

**Figure 1:**
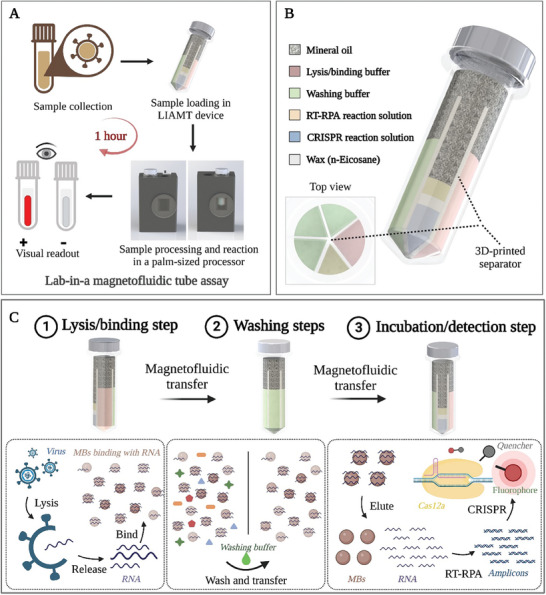
Overview of the LIAMT assay. A) Clinical assay workflow of the LIAMT platform. The LIAMT device can accept raw clinical samples. The nucleic acid sample is prepared and transferred by magnetofluidic operation in the magnetic separation well of the processor. After magnetofluidic‐based nucleic acid extraction, the LIAMT device is transferred into the incubation/detection well for isothermal amplification and CRISPR‐based fluorescence detection. The endpoint fluorescence signal of the device can be visually observed by the naked eye or recorded by a smartphone. B) Schematic illustration of the LIAMT device. The device contains five independent zones with pre‐stored lysis/binding buffer, washing buffer, and RT‐RPA/wax/CRISPR reaction solution, respectively. All buffer solutions are covered and sealed by mineral oil. C) Schematic shows the processing of clinical sample detection by the magnetofluidic separation operation in the LIAMT platform. This includes three main steps: 1) adding the clinical sample into the LIAMT device and mixing with lysis/binding buffer containing magnetic beads, 2) washing the magnetic beads bound with nucleic acids in washing buffer zones to purify nucleic acids, and 3) transferring the magnetic beads with nucleic acids into the RT‐RPA/wax/CRISPR zone for isothermal amplification and CRISPR detection.

**Figure** **1**A summarizes the clinical assay workflow of the LIAMT platform. The LIAMT platform consists of a disposable, all‐in‐one LIAMT device and a palm‐sized processor (Figure 1A). The LIAMT device can directly accept raw clinical samples (e.g., swab, plasma) and perform “sample‐to‐result” molecular diagnostics within ≈1 h. For clinical testing, the collected sample is first added to the LIAMT device. Then, the device is inserted into the magnetic separation well of the palm‐sized processor for magnetofluidic‐based nucleic acid purification. After nucleic acid purification, the device is transferred into the incubation/detection well of the processor for isothermal amplification and CRISPR detection. Last, the endpoint fluorescence signal of the device can be visually read by the naked eye or recorded by a smartphone, eliminating the need for time‐consuming nucleic acid sample preparation and expensive instrumentation (Figure 1A).

Figure 1B shows a schematic illustration of the fully integrated and self‐contained LIAMT device, which is capable of performing nucleic acid sample preparation, isothermal amplification, CRISPR cleavage reaction, and fluorescence detection in a single engineered tube. The disposable LIAMT device is composed of a 3D‐printed separator and a commercially available 1.5 mL microcentrifuge tube. The separator isolates the microcentrifuge tube into five independent zones: i) one lysis/binding buffer zone for viral lysis and nucleic acid binding on the magnetic beads, ii) three washing buffer zones for nucleic acid purification, and iii) one RT‐RPA/wax (n‐Eicosane)/CRISPR reaction zone (inset of Figure 1B). All buffer solutions and reagents are pre‐stored in the LIAMT device and covered by mineral oil. As shown in Figure 1C, the virus is first lysed to release nucleic acids when the clinical sample is mixed with the pre‐stored lysis/binding buffer in the lysis/binding buffer zone. Due to the high salt condition of the lysis/binding buffer, the released nucleic acid can bind the surface of the silica‐coated magnetic beads due to electrostatic interactions. Then, the magnetic beads bound with nucleic acids are transferred to the washing buffer zones to remove potential inhibitors by the magnetofluidic separation approach. Next, the magnetic beads carrying nucleic acids are transferred to the RT‐RPA/wax/CRISPR reaction zone for isothermal amplification and CRISPR‐based fluorescence detection. In this reaction solution, the nucleic acid molecules will be released from the surface of the magnetic beads due to the low salt condition of the reaction buffer. Thus, the LIAMT platform provides a simple, rapid, and affordable “sample‐to‐result” solution to detect virus in clinical samples at the point‐of‐care.

### 2.2 Optimization of Magnetofluidic‐Based Nucleic Acid Sample Preparation

**Figure 2:**
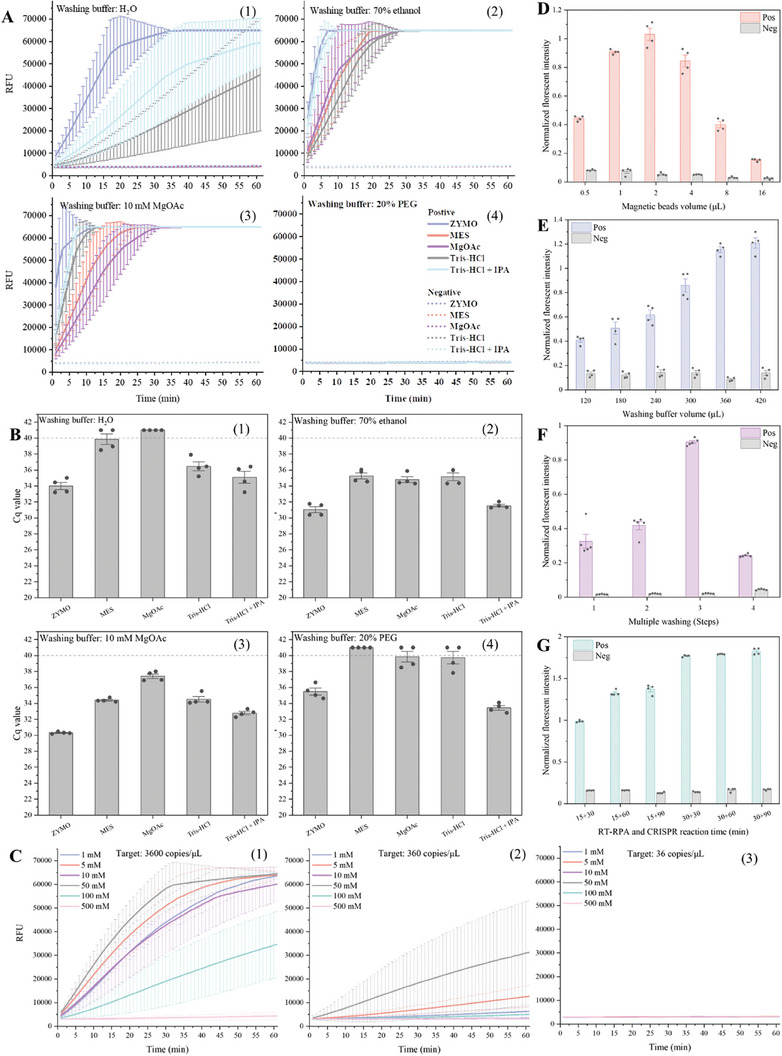
Optimization of magnetofluidic‐based nucleic acid detection. A,B) Comparison and optimization of five lysis/binding buffer solutions (ZYMO, MES, MgOAc, Tris‐HCl, and Tris‐HCl+IPA) and four washing buffer solutions (H_2_O, 70% ethanol, 10 mm MgOAc with 1% Triton X‐100, and 20% PEG solution) by RT‐RPA/CRISPR and RT‐PCR, respectively. C) Six different concentrations (1, 5, 10, 50, 100, and 500 mm) of MgOAc solution with 1% Triton X‐100 were prepared and tested as washing buffer in the LIAMT device with various SARS‐CoV‐2 concentrations (3,600, 360, and 36 copies µL^−1^). The real‐time fluorescence curves were recorded by a real‐time PCR machine. D) Effect of the magnetic bead volume (0.5, 1, 2, 4, 8, and 16 µL) on the magnetofluidic‐based nucleic acid extraction. E) Effect of the washing buffer volume ranging from 120 to 420 µL on the magnetofluidic‐based nucleic acid extraction. F) Effect of the number of washing steps on the magnetofluidic‐based nucleic acid detection. G) Comparison and optimization of the incubation time for the RT‐RPA reaction and CRISPR reaction, respectively. The endpoint fluorescence signal was detected by ChemiDoc MP Imaging System.

To prepare high‐quality nucleic acid samples, we compared and optimized different lysis/binding buffer and washing buffer solutions for the magnetofluidic‐based nucleic acid extraction. First, we evaluated and compared five different lysis/binding buffer solutions, including: i) ZYMO lysis/binding buffer (ZYMO Research), ii) MES buffer, iii) MgOAc buffer, iv) Tris‐HCl buffer, and v) Tris‐HCl + isopropanol buffer^[^ ^18^, ^26^, ^27^ ^]^. In addition, we evaluated four different washing buffer solutions, including: i) deionized water, ii) 70% ethanol, iii) MgOAc with 1% Triton X‐100, and iv) 20% polyethylene glycol (PEG) solution^[^ ^18^, ^26^ ^]^. As shown in **Figure** **2**A, the combination of the ZYMO lysis/binding buffer and MgOAc washing buffer with 1% Triton X‐100 showed the best performance in the RT‐RPA/CRISPR assay. To further validate its versatility for other nucleic acid amplification testing, we tested the extracted nucleic acids by using the RT‐PCR assay and obtained similar results as the RT‐RPA/CRISPR assay (Figure 2B). Next, we further evaluated the effect of the MgOAc concentration in the washing buffer on the nucleic acid extraction and downstream amplification detection. We compared and tested six different concentrations (1, 5, 10, 50, 100, and 500 mm) of MgOAc buffer with 1% Triton X‐100 by various SARS‐CoV‐2 concentration samples in our LIAMT device. To monitor the fluorescence signal of the CRISPR reaction in real time, we removed the RPA amplicons of each tested sample from the device and added them to the CRISPR reaction tubes for real‐time fluorescence monitoring. As shown in Figure 2C, the washing buffer of 50 mm MgOAc consistently showed the highest fluorescence signal for SARS‐CoV‐2 detection. Thus, we determined the optimal lysis/binding buffer and washing buffer for our LIAMT device to be ZYMO lysis/binding buffer and 50 mm MgOAc washing buffer with 1% Triton X‐100, respectively.

Unlike conventional magnetic bead‐based nucleic acid extraction methods^[^ ^28^, ^29^, ^30^ ^]^, our LIAMT assay retains the magnetic beads in the RT‐RPA reaction solution, eliminating the need for an additional elution step. Thus, the usage amount of the magnetic beads can affect the downstream nucleic acid detection. To this end, we tested a series of volumes of magnetic beads ranging from 0.5 to 16 µL in the LIAMT device. We determined the optimal volume of the magnetic beads to be 2 µL, as it led to the strongest fluorescence signals (Figure 2D). Interestingly, as the usage of the magnetic beads increased, there was a significant reduction in the fluorescence signals of the device. This result could be attributed to two major factors: i) the increased amount of magnetic beads may introduce more potential inhibitors into the downstream RT‐RPA reaction solution, and ii) an excessive amount of magnetic beads can potentially interfere with the RT‐RPA reaction and reduce its amplification efficiency. In addition, we evaluated the effect of the washing buffer volume and washing times on the LIAMT assay. We tested various volumes of the washing buffer ranging from 120 to 420 µL. As expected, increasing the volume of the washing buffer resulted in a higher fluorescence detection signal (Figure 2E). Considering the limited volume capacity of our LIAMT device, we used 420 µL of washing buffer to wash the magnetic beads and purify the nucleic acids. To optimize the washing efficiency, we further investigated the number of washing steps by evenly dividing 420 µL of washing buffer into 1, 2, 3, and 4 equal parts. As we increased the number of washing times, we observed an increased fluorescence signal (Figure 2F). However, when we applied more than three washing steps, the detection signals dramatically reduced, which may be attributed to the increased loss of magnetic beads during the magnetofluidic transferring process. Therefore, we used 2 µL of magnetic beads and 420 µL of washing buffer with three washing steps in the design of our LIAMT device for the subsequent experiments.

Last, we determined the effect of the incubation time of the RT‐RPA reaction and CRISPR reaction for nucleic acid amplification and detection. To maximize the efficiency of the RT‐RPA reaction, the RT‐RPA preamplification independently occurs in the RT‐RPA/wax/CRISPR zone of the device. This separation occurs because its reaction mixture is initially isolated by the wax from the CRISPR reaction solution. Thus, we compared and tested their respective reaction times. As shown in Figure 2G, a 30 min RT‐RPA preamplification reaction and 30 min CRISPR reaction generated the strongest fluorescence signal for detection. Increasing the CRISPR reaction time did not significantly improve the fluorescence signal. Thus, we chose to use a 30 min RT‐RPA preamplification reaction and 30 min CRISPR reaction for our LIAMT device.

### 2.3 Design and Operation of the LIAMT Device

**Figure 3:**
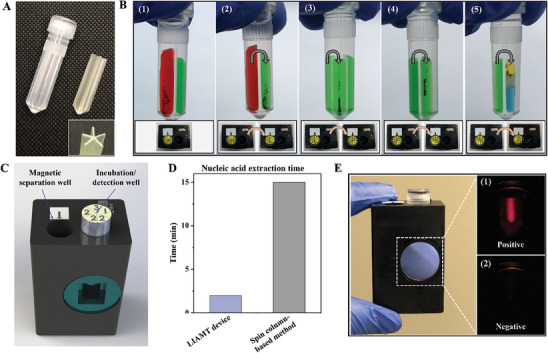
LIAMT platform and its operation. A) Photograph of the LIAMT device and its 3D‐printed separator. Inset is the top view of the separator. B) Magnetofluidic‐based nucleic acid extraction steps in the LIAMT device. 1) Clinical sample is added into the device and mixed with the lysis/binding buffer containing magnetic beads. The nucleic acids are released from virus and bound on the magnetic beads. The magnetic beads in the lysis/binding buffer zone (as indicated by “1” on the cap) are captured and transferred to the mineral oil layer by the neodymium magnet (as indicated by the arrow in the processor) when the device is inserted into the magnetic separation well. 2) The LIAMT device is rotated clockwise to the first washing buffer zone (as indicated by “2” on the cap) and then lifted for the first washing step. 3 & 4) The magnetic beads are washed two more times by repeating the magnetofluidic separation operation. 5) The magnetic beads bound with nucleic acids are transferred into the RPA/wax/CRISPR reaction zone. C) Schematic illustration of the palm‐sized processor, which contains a magnetofluidic separation well for magnetofluidic‐based nucleic acid extraction, and an incubation/detection well for isothermal amplification and CRISPR detection. D) Comparison of nucleic acid sample preparation time between the traditional spin column‐based method and the magnetofluidic method of the LIAMT device. E) Photograph of the palm‐sized processor. Endpoint fluorescence images of 1) SARS‐CoV‐2‐positive and 2) SARS‐CoV‐2‐negative samples.

After optimizing the experimental conditions of the magnetofluidic‐based nucleic acid detection, we designed, fabricated, and tested a disposable, all‐in‐one, LIAMT device for “sample‐to‐result” detection of virus. We used 3D‐printing technology to fabricate our separator (**Figure** **3**A) because of its low cost and ease of use^[^ ^31^, ^32^, ^33^ ^]^. To minimize liquid leakage, we optimized the design of the 3D‐printed separator and improved its mechanical strength by using UV curing (Figure S1, Supporting Information). Furthermore, we coated polydimethylsiloxane (PDMS) solution on the edges of the separator to enable a seamless seal between the separator and microcentrifuge tube. For clinical testing, we directly add the raw clinical samples into the lysis/binding buffer zone to lyse the virus and release nucleic acids (Figure 3B). Thus, the released nucleic acid binds the surface of the silica‐coated magnetic beads due to electrostatic interactions. After inserting the device into the magnetic separation well of the processor (Figure 3C), the magnetic beads are transferred to the washing buffer zones for nucleic acid purification by manually rotating and lifting the device (Video S1, Supporting Information). To minimize the potential inhibitor's interference and obtain high‐quality nucleic acids, we designed the separator with three buffer zones to wash the magnetic beads bound with nucleic acids according to our optimized experimental conditions above (Figure 2F). The nucleic acid extraction efficiency of the LIAMT device was 75 ± 12% (n  =  3), which was comparable to conventional nucleic acid extraction (76 ± 8%, n  =  3). The total time required for magnetofluidic‐based nucleic acid extraction in the LIAMT device is estimated to be ≈2 min, which is ≈8 times faster than that of the conventional nucleic acid extraction (i.e., ≈15 min) (Figure 3D).

After the magnetic beads are transferred into the RT‐RPA/wax/CRISPR reaction zone, the LIAMT device is inserted into the incubation/detection well for isothermal amplification and CRISPR detection (Figure 3E). The nucleic acids bound on the magnetic beads release into the RT‐RPA reaction mixture and directly serve as the template of the RT‐RPA amplification reaction when the processor elevates the temperature to ≈35 °C. At 35 °C, n‐Eicosane wax maintains a solid state because its melting point is typically 36–38 °C. After a 30 min preamplification, the temperature of the processor is raised to above 40 °C, which melts the n‐Eicosane wax. With the melting of this wax layer, the RPA amplification product is combined with the CRISPR reaction solution, resulting in specific activation of the CRISPR‐Cas12a enzymes in the presence of nucleic acid target. Thus, the activated Cas12a enzyme non‐specifically cleaves the cyanine‐5 dye (Cy5)/quencher‐labeled single‐stranded DNA fluorescence probe (ssDNA‐Cy5/quencher), generating a strong fluorescence signal for visual detection (see inset of Figure 3E).

### 2.4 Detection of Viral RNA by the LIAMT Platform

To enable point‐of‐care diagnostic applications, we designed and fabricated a portable, palm‐sized processor (40 mm × 30 mm × 60 mm) (Figure 3C,E; Figure S2, Supporting Information) matched with the disposable LIAMT device, eliminating the need for expensive equipment. The processor can be powered by a portable charger. The palm‐sized processor features three major functions: i) facilitating magnetic bead separation and transferring by magnetofluidic operation, ii) incubating the LIAMT device at desired temperatures for the RT‐RPA reaction and CRISPR cleavage, and iii) enabling visual endpoint fluorescence detection (Figure S1, Supporting Information). The palm‐sized processor contains two functional wells: i) a magnetic separation well containing a neodymium magnet (as indicated by the arrow in the processor) (Figure 3C), designed for the magnetofluidic operation and magnetic bead separation, and ii) an incubation/detection well, for RT‐RPA/CRISPR reaction incubation and visual fluorescence detection. To incubate the LIAMT device for RT‐RPA/CRISPR, we attached a thin‐film heater on the wall of the incubation/detection well (Figure S2, Supporting Information). To observe the endpoint fluorescence, we used a 645‐nm LED as the excitation light source and Cy5 fluorescence filter set for fluorescence detection (Figure S2, Supporting Information).

**Figure 4:**
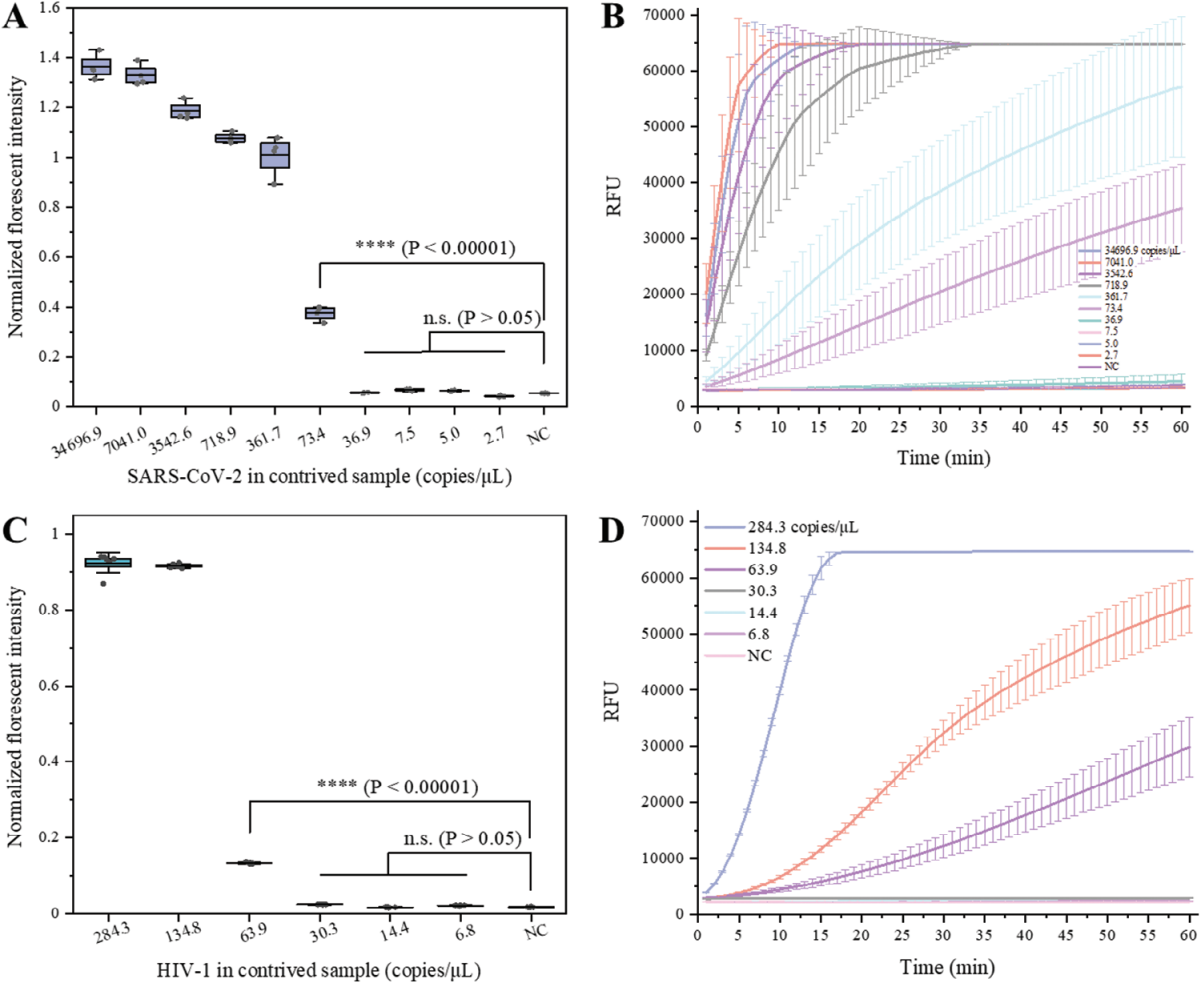
Virus detection in spiked samples by using the LIAMT platform. A) Normalized fluorescence intensity of the device for SARS‐CoV‐2 detection. NC, negative control. n.s., not significant with *p* > 0.05. ^****^ *p* ≤0.00001. B) Real‐time fluorescence detection of the CRISPR reaction after separate RT‐RPA preamplification of SARS‐CoV‐2 in the spiked samples. n = 3. C) Normalized fluorescence intensity for HIV detection in the LIAMT device. D) Real‐time fluorescence detection of RPA/CRISPR reaction of HIV in the plasma samples. n = 3.

We evaluated the detection sensitivity of the LIAMT device using serial dilutions of virus in the spiked samples. To enable accurate quantitative evaluation, we first detected and quantified the copy number of SARS‐CoV‐2 and HIV in the samples by QuantStudio 3D digital PCR chips (Figures S3 and S4, Supporting Information, respectively). Then, we tested serial dilutions of virus in spiked samples by using the LIAMT device. As shown in **Figure** **4**A, the higher the SARS‐CoV‐2 concentration, the stronger the observed endpoint fluorescence signal. With the LIAMT platform, we could consistently detect 73.4 copies µL^−1^ of SARS‐CoV‐2 RNA, which is comparable to conventional RT‐RPA/CRISPR detection using a real‐time PCR machine (Figure 4B). Additionally, we detected HIV in plasma samples and achieved a sensitivity of 63.9 copies µL^−1^ (Figure 4C), which is consistent with that of the RT‐RPA/CRISPR assay (Figure 4D). Thus, our LIAMT platform provides a simple, reliable, and sensitive approach for virus detection at the point‐of‐care without the need for complex equipment.

### 2.5 Clinical Validation of the LIAMT Assay Platform

**Figure 5:**
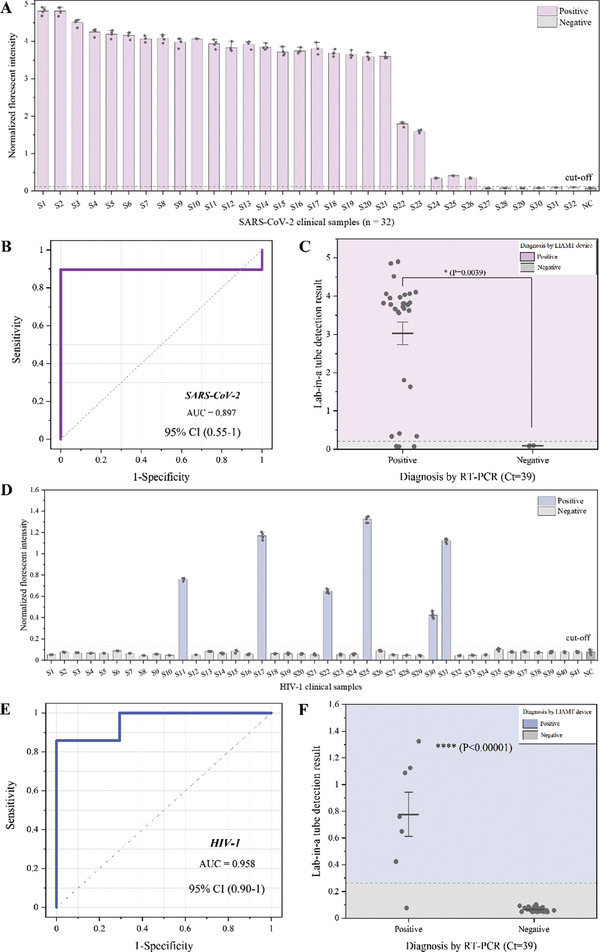
Clinical validation of the LIAMT platform to detect SARS‐CoV‐2 and HIV in clinical samples. A) Normalized fluorescence intensity of the LIAMT device for SARS‐CoV‐2 detection in 32 clinical swab samples. B) ROC curve of the LIAMT results for SARS‐CoV‐2 detection. ROC curve analysis of the device compared to the standard RT‐PCR method. C) Statistical analysis of the LIAMT results for SARS‐CoV‐2 detection. D) Normalized fluorescence intensity of the LIAMT device for HIV detection in 41 clinical plasma samples. E) ROC curve of the LIAMT results for HIV detection. F) Statistical analysis of the LIAMT results for HIV detection.

To further validate the clinical utility of our LIAMT platform, we conducted a feasibility study with 73 clinical samples, including 32 clinical nasopharyngeal swab samples for SARS‐CoV‐2 and 41 clinical plasma samples for HIV. Before testing these samples with the LIAMT platform, we performed real‐time RT‐PCR to confirm the clinical samples. In clinical diagnostics by PCR/RT‐PCR^[^ ^3^, ^34^, ^35^, ^36^ ^]^, the accepted cut‐off for the quantification cycle (Cq) value ranges from 35–40. Typically, samples with Cq > 35 are considered to have a low viral load. Here, we used a cutoff Cq value of 39 to determine the sample positivity. As shown in Figure S5 (Supporting Information), among the 32 clinical samples, 29 samples (Samples S1–S29, Supporting Information) were determined to be positive, and three samples (Samples S30–S32, Supporting Information) were determined to be negative. We next tested these clinical samples by using the LIAMT platform, capturing end‐point fluorescence images by a smartphone (Figure S6, Supporting Information). To further analyze the fluorescence images, we used ImageJ software to quantify the fluorescence intensity of the images (**Figure** **5**A). We normalized the fluorescence intensity and set the cut‐off threshold to 0.12, which was calculated by µ ± 3σ, where µ is the mean value of the negative control and σ is the standard deviation. Although our LIAMT device is not intended for quantitative detection, we found that lower Cq values of the RT‐PCR assay in the clinical samples resulted in stronger fluorescence intensities in the device (Figure 5A). With our LIAMT platform, we could distinguish 26 samples as positive from all 32 clinical samples, consistent with that of the conventional RT‐RPA/CRISPR detection (Figure S7, Supporting Information). However, both the LIAMT platform and conventional RT‐RPA/CRISPR assay failed to detect three samples (Samples S27–S29, Supporting Information, with Cq values of 38.05, 38.33, and 38.22, respectively) as positive. In our previous study^[^ ^3^ ^]^, samples with Cq values close to 39 were either weak or false positives. Finally, we assessed the receiver operating characteristic (ROC) of the LIAMT platform and performed a statistical analysis. The LIAMT platform obtained an area under the curve (AUC) of 0.897 (95% confidence interval [CI], 0.55–1) for SARS‐CoV‐2 detection in clinical samples (Figure 5B). Additionally, the positive samples showed an elevated fluorescence signal compared to the negative samples (Figure 5C).

To further evaluate the versatility of the developed LIAMT platform, we tested 41 clinical plasma samples for HIV. Of the 41 clinical samples, seven were determined by RT‐PCR as positive for HIV and 34 as negative (Figure S8, Supporting Information). Detecting these clinical samples by the LIAMT platform could distinguish all six positive samples except for sample 39 (Figure 5D; Figure S9, Supporting Information), consistent with that of the two‐step RT‐RPA/CRISPR method (Figure S10, Supporting Information). Sample 39 has a Cq value of 38.97, which is close to the cutoff Cq value of 39. To evaluate the assay performance, we next performed an ROC curve analysis and statistical analysis on the LIAMT clinical results (Figure 5E,F). The LIAMT platform showed excellent performance (AUC = 0.958, 95% CI, 0.90–1). Therefore, our simple, affordable, and portable LIAMT platform provides a promising diagnostic alternative for virus detection as a versatile diagnostic tool at the point‐of‐care.

## 3 Conclusion

In this study, we developed a simple, affordable, portable, and sensitive LIAMT platform for “sample‐to‐result” virus detection at the point‐of‐care. The LIAMT platform contains a disposable LIAMT device and a palm‐sized processor. The LIAMT device integrates virus lysis, RNA extraction, RT‐RPA amplification, and CRISPR‐Cas12a cleavage reaction in a single tube. The palm‐sized processor has multiple functions, including magnetofluidic separation, incubating the device, and enabling visual fluorescence detection. The LIAMT platform can detect SARS‐CoV‐2 and HIV with a sensitivity of 73.4 and 63.9 copies µL^−1^, respectively, within ≈1 h. To demonstrate its clinical utility, we used the LIAMT platform to detect both SARS‐CoV‐2 and HIV in clinical samples, achieving a comparable performance to that of the real‐time RT‐PCR approach. Compared with other research (Table S1, Supporting Information)^[^ ^30^, ^34^, ^37^, ^38^, ^39^, ^40^, ^41^, ^42^ ^]^, our LIAMT platform offers several advantages: i) “Sample‐to‐result” nucleic acid‐based molecular detection. By taking advantage of the magnetofluidic approach to manipulate magnetic beads, the LIAMT platform is capable of performing nucleic acid sample preparation, isothermal amplification, and CRISPR detection in a simple engineered microcentrifuge tube. ii) Palm‐sized processor enabling point‐of‐care diagnostics. We developed a palm‐sized, multifunctional processor for magnetofluidic operation, isothermal amplification, and fluorescence detection, eliminating the need for expensive equipment and enabling point‐of‐care diagnostic applications. iii) Simplicity and portability. We used a simple 3D‐printing technology to fabricate the separator and repurpose the disposable microcentrifuge tube, enabling the development of a low‐cost, portable LIAMT device (<$5). We pre‐stored all reagents and buffer solutions in the device, eliminating the need for complex reagent transferring and manual operation. In the future, we will integrate an automatic magnetofluidic operation into the processor to enhance the automation of the LIAMT assay^[^ ^43^ ^]^. In addition, we will further lyophilize the reagents in the LIAMT device to simplify the device operation^[^ ^44^, ^45^ ^]^. Overall, our simple, portable, sensitive, and self‐contained LIAMT platform is a promising tool for rapid detection of infectious disease at the point‐of‐care, especially in resource‐limited settings.

## 4 Experimental Section

### Reagent Preparation for the LIAMT Assay

The reagents of the LIAMT assay include lysis/binding buffer, washing buffer, RT‐RPA reaction solution, and CRISPR Cas12a reaction mixture. The lysis/binding buffer (D7020‐1‐100) was obtained from the Quick‐DNA/RNA Viral MagBead extraction kit (R2140‐E, ZYMO Research, USA). The washing buffer is a 50 mm MgOAc (CAS: 16674‐78‐5, Sigma–Aldrich) solution with 1% Triton X‐100 (CAS: 9036‐19‐5, Sigma–Aldrich). The RPA kit, TwistAmp Basic, was obtained from Twist Dx Limited (Maidenhead, UK). RNase H and nuclease‐free water were purchased from New England BioLabs (MA, USA). SuperScript IV Reverse Transcriptase was purchased from ThermoFisher Scientific (MA, USA). EnGen Lba Cas12a was purchased from New England BioLabs (MA, USA). CRISPR RNA (crRNA) and fluorescent reporter (ssDNA‐Cy5/quencher) were synthesized from Integrated DNA Technologies (Tables S2 and S3, Supporting Information).

The RT‐RPA/wax/CRISPR reaction zone contains 50 µL of RT‐RPA reaction mixture, 20 µL of n‐Eicosane wax, and 60 µL of CRISPR Cas12a reaction solution. A total of 60 µL of CRISPR Cas12a reaction mixture was prepared, containing 400 nm Lba Cas12a, 400 nm crRNA, 1X NEBuffer 2.1 (50 mm NaCl, 10 mm Tris‐HCl, 10 mm MgCl_2_, 100 µg mL^−1^ BSA, pH 7.9, 25 °C), and 4 µm ssDNA‐Cy5/quencher fluorescent reporter. The prepared CRISPR mixture was pre‐stored in the device and sealed with 20 µL of n‐Eicosane wax (CAS: 112‐95‐8, Sigma–Aldrich). The RT‐RPA reaction mixture (in 50 µL) contained one dried pellet, 29.5 µL of primer‐free rehydration buffer, 0.5 µm each for forward and reverse primers, 2 U µL^−1^ Superscript IV reverse transcriptase, 0.1 U µL^−1^ RNase H, and 14 mm MgOAc. In the LIAMT assay, RT‐RPA reaction mixture was directly added on the top of the wax and formed an RT‐RPA/wax/CRISPR reaction system.

### LIAMT Device Design and Fabrication

The LIAMT device consists of a 3D‐printed separator and a microcentrifuge tube (1.5 mL conical screw cap tubes, 02‐681‐373, Fisher Scientific, NH, USA). The separator was designed using Solidworks software and fabricated by a Form 3 3D printer using clear resin (RS‐F2‐GPCL‐04, Formlabs, MA, USA). The separator divided the microcentrifuge tube into five independent zones, including a lysis/binding buffer zone (with a 90° angle), three wash zones (with a 70° angle each), and an RT‐RPA/wax/CRISPR detection zone (with a 60° angle) (as shown in the insets of Figures 1B and 3A). The different thickness of the 3D printed separator to optimize its design was evaluated and compared. Next, the 3D‐printed separator was washed with isopropyl alcohol for at least 30 min and cured under UV light for at least 3 h. After UV curing, the edges of the separator were coated with polydimethylsiloxane (PDMS) solution (RTV615 001‐KIT, Momentive Performance Materials, NY, USA). Then, the 3D‐printed separator coated with PDMS solution was inserted into a microcentrifuge tube. The sealing with PDMS ensures no liquid leakage between different buffer zones. Last, the assembled LIAMT devices were placed at room temperature for more than 12 h, allowing the PDMS to completely solidify.

### Portable Palm‐Sized Processor Development

The palm‐sized processor was designed using Solidworks and fabricated by a Form 3 3D printer using black resin (RS‐F2‐GPBK‐04, Formlabs, MA, USA). The processor has two vial wells: i) one magnetofluidic separation well for magnetofluidic transferring and operation, and ii) one incubation/detection well for RT‐RPA/CRISPR incubation and fluorescence detection (Figure 3C). In the magnetofluidic separation well, a cylindrical neodymium magnet (6.33 mm diameter × 6.33 mm height) was embedded to separate the magnetic beads and transfer them into different functional zones of the device through magnetic force. In the incubation/detection well, a flexible thin‐film heater (HK5572R26.5L23A, Minco, MN, USA) and a thermocouple wire were fixed on its inner wall to heat the device and monitor the temperature, respectively (Figure S2, Supporting Information).

To visualize the fluorescence signal of the LIAMT device, a Cy5 fluorescence filter set (#67‐010, Edmund Optics, NJ, USA) along with LED lights (LED645L, Thorlabs, NJ, USA) were used to build an optical detection path. The LED was placed at the bottom of the incubation/detection well and served as an excitation light source (Figure S2, Supporting Information). To block other wavelength light, the LED light was filtered by an excitation filter (#67‐035 in Cy5 fluorescence filter set, wavelength 604 – 644 nm). To observe specific emission fluorescence signal from the LIAMT device, the emission filter (#67‐038 in the Cy5 fluorescence filter set, wavelength 672–712 nm) was placed on the observation window of the processor (Figure S2, Supporting Information).

### Operation and Detection of the LIAMT Platform

For clinical testing with the LIAMT device, 60‐µL clinical samples were added into the lysis/binding buffer zone containing pre‐stored lysis/binding buffer (120 µL) and magnetic beads (2 µL) (Quick‐DNA/RNA Viral MagBead extraction kit). After samples were introduced and mixed with the lysis/binding buffer, mineral oil (#1632129, Bio‐Rad, USA) was added into the LIAMT device to cover all buffer solutions, thereby completely isolating the individual zones. Then, the device was sealed by a screw cap marked by yellow tape, indicating the location of the different zones (Figure 3B,C). The numbers 1, 2, and 3 on the screw cap show the lysis/binding buffer zone, the washing buffer zone, and the RT‐RPA/wax/CRISPR reaction zone, respectively (Figure 3B,C). The location of the neodymium magnet was marked with an arrow in the magnetofluidic separation well of the processor (Figure 3B,C). And then, the device was slowly inserted into the magnetofluidic separation well by aligning its lysis/binding zone (as indicated by “1” on the cap) with the magnet (as indicated by the arrow in the processor), the magnetic beads were collected and moved into the mineral oil layer by magnetic force. Then, the LIAMT device was slowly rotated clockwise to the first washing buffer zone (as indicated by “2” on the cap) and was manually lifted, which brought the magnetic beads into the first washing buffer for purification. After two more washing steps by the same operations, the magnetic beads bound with nucleic acids were transferred into the RT‐RPA/wax/CRISPR reaction zone. Last, the LIAMT device was inserted into the incubation/detection well of the palm‐sized processor for the RT‐RPA reaction and CRISPR detection. The thin‐film flexible heater first heated the device for the RPA preamplification reaction at ≈35 °C, in which the n‐Eicosane wax remains solid and isolates the RT‐RPA reaction mixture and CRISPR reaction solution. After a 30 min RPA preamplification, the heater's temperature was elevated to ≈40 °C. The elevated temperature resulted in the melting of the n‐Eicosane wax, which brought the RT‐RPA preamplification reaction mixture and CRISPR reaction solution together and initiated the CRISPR detection. After a 30 min CRISPR reaction, the fluorescence signal of the CRISPR reaction was visually observed by the naked eye through the observation window on the processor (Figure 3E).

### Selection and Optimization of Lysis/Binding Buffer and Washing Buffer

Five lysis/binding buffer solutions, including: i) ZYMO lysis/binding buffer (R2140‐E, ZYMO Research, USA), ii) MES binding buffer (4 m guanidine thiocyanate (GuSCN), 10 mm MES [2‐ethanesulfonic acid], 1% Triton X‐100, 1% β‐mercaptoethanol), iii) MgOAc lysis/binding buffer (5 m guanidine thiocyanate, 100 mm MgOAc, 1% Triton X‐100), iv) Tris‐HCl lysis/binding buffer (4 m guanidine thiocyanate, 55 mm Tris HCl pH 7.5, 25 mM EDTA, and 3% Triton X‐100), and v) Tris‐HCl with isopropanol binding buffer (50% v/v Tris‐HCl binding buffer and 50% v/v isopropanol) are compared and tested. In addition, four washing buffer solutions were prepared and tested, including: i) deionized water, ii) 70% ethanol, iii) MgOAc with 1% Triton X‐100, and iv) 20% PEG solution. For the optimization of MgOAc washing buffer, six MgOAc washing buffer solutions with various concentrations (1, 5, 10, 50, 100, and 500 mm with 1% Triton X‐100) were prepared and tested. To optimize the usage of the magnetic beads and washing buffer, serial volumes of magnetic beads (0.5, 1, 2, 4, 8, and 16 µL) and washing buffer (120, 180, 240, 300, 360, and 420 µL) were prepared and tested. Additionally, different washing operations (e.g., 1, 2, 3, and 4 washing steps) were evaluated and compared in the LIAMT device.

### Preparation and Detection of Spiked Samples and Clinical Samples

Before detection on clinical samples, swab samples spiked with heat‐inactivated SARS‐CoV‐2 virus (Isolate USA‐WA1/2020, BEI, USA) were used to evaluate the analytical performance of the LIAMT device. For HIV detection, AcroMetrix HIV‐1 High Control (Thermo Fisher) was used to prepare spiked plasma samples. Before testing, the copy numbers of the SARS‐CoV‐2 and HIV in the samples were quantified by digital RT‐PCR. De‐identified nasopharyngeal swab samples were obtained and tested for SARS‐CoV‐2 and clinical plasma samples for HIV under the approval of the Institutional Review Board of the University of Connecticut Health Center (protocol #P61067). For RT‐PCR or RT‐RPA/CRISPR detection, the viral RNA samples were extracted using the QIAamp Viral RNA Mini Kit (Qiagen) according to the manufacturer's protocol.

### RT‐PCR and RT‐RPA/CRISPR Detection

A real‐time fluorescence RT‐PCR assay was used to detect the extracted RNA as the gold‐standard method. The GoTaq Probe 1‐Step RT‐qPCR kit from Promega (WI, USA) was used to prepare the RT‐PCR reaction solution. A total 20 µL of RT‐PCR mix was used, including 1x GoTaq Probe Master Mix, 1x GoScript RT Mix for 1‐Step RT‐qPCR, 0.5 µm forward primer, 0.5 µm reverse primer, 0.2 m probe, and 2 µL of the target. The RT‐PCR protocol contains three steps: i) reverse transcription (15 min at 45 °C), ii) RT inactivation and polymerase activation (2 min at 95 °C), and iii) denaturation and extension (40 cycles of 15 s at 95 °C and 60 s at 60 °C). Real‐time RT‐PCR detection was performed using the Bio‐Rad CFX96 Touch Real‐Time PCR Detection System.

For comparison purpose with the LIAMT device, the conventional RT‐RPA/CRISPR was applied to test the extracted RNA in the samples. For the RT‐RPA reaction, the extracted RNA from the samples was added to the RT‐RPA reaction mixture. After the RT‐RPA reaction, 2 µL of RT‐RPA amplification product was introduced to the CRISPR reaction mixture for CRISPR detection. ssDNA‐FQ probe was used for the real‐time CRISPR fluorescence detection by a real‐time PCR machine.

### Digital PCR Quantification of Target

BioRad Reliance One‐Step Multiplex RT‐qPCR Supermix (Cat. #12010176) was purchased from BioRad. The RT‐PCR mixture was prepared according to the manufacturer's protocol. After 14.5 µL of reaction mixture was loaded into the 3D Digital PCR chip (Cat. #A26316) by using a 3D Digital PCR Chip Loader (Cat. #A29154), the chip was placed on a ProFlex 2x Flat Block Thermal Cycler (Cat. #A26316) for the PCR reaction. At the end of the RT‐PCR reaction, the chip was read on either a QuantStudio 3D Digital PCR Instrument (Cat. #A26316) or a fluorescence microscope Axio Observer from ZEISS.

### Statistical Analysis

Statistical analyses were performed by OriginLab 2020. All statistical analyses were performed using multiple Student's T‐test, where n.s. = not significant with *p* > 0.05, and asterisks (^*^, ^**^, ^***^, ^****^) denoting significant differences with the following *p* values (^*^ = 0.001 < *p* ≤0.05, ^**^ = 0.0001 < *p* ≤0.001, ^***^ = 0.00001 < *p* ≤0.0001, ^****^ = *p* ≤0.00001).

## Conflict of Interest

The authors declare no conflict of interest.

## Author Contributions

Z.L. and C.L. conceived the idea. Z.L., S.Z., J.Z., and L.A. carried out the experiments. Z.L., S.Z., J.Z., D.B., H.Z., and C.L. analyzed the data. C.L. supervised the project. Z.L. and C.L. wrote the manuscript. All authors discussed the results and commented on the manuscript.

## Supporting information

Supporting Information

Supplemental Video 1

## Data Availability

The data that support the findings of this study are available from the corresponding author upon reasonable request.

## References

[1] J. Song , B. Cha , J. Moon , H. Jang , S. Kim , J. Jang , D. Yong , H.‐J. Kwon , I.‐C. Lee , E.‐K. Lim , J. Jung , H. G. Park , T. Kang , ACS Nano 2022, 16, 11300. 35735410 10.1021/acsnano.2c04840 PMC9236205

[2] N. N. Y. Tsang , H. C. So , K. Y. Ng , B. J. Cowling , G. M. Leung , D. K. M. Ip , Lancet Infect. Dis. 2021, 21, 1233. 33857405 10.1016/S1473-3099(21)00146-8 PMC8041361

[3] Z. Li , N. Uno , X. Ding , L. Avery , D. Banach , C. Liu , ACS Nano 2023, 17, 3966. 36762838 10.1021/acsnano.2c12754 PMC10198471

[4] F. Shen , B. Sun , J. E. Kreutz , E. K. Davydova , W. Du , P. L. Reddy , L. J. Joseph , R. F. Ismagilov , J. Am. Chem. Soc. 2011, 133, 17705. 21995644 10.1021/ja2060116 PMC3216675

[5] R. Nouri , Y. Jiang , A. J. Politza , T. Liu , W. H. Greene , Y. Zhu , J. J. Nunez , X. Lian , W. Guan , ACS Nano 2023, 17, 10701. 37252938 10.1021/acsnano.3c01917 PMC11240847

[6] M. Mauk , J. Song , H. H. Bau , R. Gross , F. D. Bushman , R. G. Collman , C. Liu , Lab Chip 2017, 17, 382. 28092381 10.1039/c6lc01239f PMC5285266

[7] M. A. Dineva , L. Mahilum‐Tapay , H. Lee , Analyst 2007, 132, 1193. 18318279 10.1039/b705672a

[8] D. Chen , M. Mauk , X. Qiu , C. Liu , J. Kim , S. Ramprasad , S. Ongagna , W. R. Abrams , D. Malamud , P. L. A. M. Corstjens , H. H. Bau , Biomed. Microdevices 2010, 12, 705. 20401537 10.1007/s10544-010-9423-4 PMC2924744

[9] X. Wang , H. Yao , X. Xu , P. Zhang , M. Zhang , J. Shao , Y. Xiao , H. Wang , Clin. Chem 2020, 66, 977. 32282874 10.1093/clinchem/hvaa099 PMC7184447

[10] T. Suo , X. Liu , J. Feng , M. Guo , W. Hu , D. Guo , H. Ullah , Y. Yang , Q. Zhang , X. Wang , M. Sajid , Z. Huang , L. Deng , T. Chen , F. Liu , K. Xu , Y. Liu , Q. Zhang , Y. Liu , Y. Xiong , G. Chen , K. Lan , Y. Chen , Emerg. Microbes Infect. 2020, 9, 1259. 32438868 10.1080/22221751.2020.1772678 PMC7448897

[11] H. Zhu , H. Zhang , Y. Xu , S. Laššáková , M. Korabečná , P. Neužil , BioTechniques 2020, 69, 317. 32815744 10.2144/btn-2020-0057 PMC7439763

[12] N. Lübke , T. Senff , S. Scherger , S. Hauka , M. Andrée , O. Adams , J. Timm , A. Walker , J. Clinical Virology 2020, 130, 104579. 32795959 10.1016/j.jcv.2020.104579 PMC7405857

[13] M. A. Lalli , J. S. Langmade , X. Chen , C. C. Fronick , C. S. Sawyer , L. C. Burcea , M. N. Wilkinson , R. S. Fulton , M. Heinz , W. J. Buchser , R. D. Head , R. D. Mitra , J. Milbrandt , Clin. Chem. 2021, 67, 415. 33098427 10.1093/clinchem/hvaa267 PMC7665435

[14] I. Smyrlaki , M. Ekman , A. Lentini , N. Rufino de Sousa , N. Papanicolaou , M. Vondracek , J. Aarum , H. Safari , S. Muradrasoli , A. G. Rothfuchs , J. Albert , B. Högberg , B. Reinius , Nat. Commun. 2020, 11, 4812. 32968075 10.1038/s41467-020-18611-5 PMC7511968

[15] C. Liu , E. Geva , M. Mauk , X. Qiu , W. R. Abrams , D. Malamud , K. Curtis , S. M. Owen , H. H. Bau , Analyst 2011, 136, 2069. 21455542 10.1039/c1an00007a PMC4360993

[16] J. Wen , L. A. Legendre , J. M. Bienvenue , J. P. Landers , Anal. Chem. 2008, 80, 6472. 18754652 10.1021/ac8014998

[17] Y.‐K. Cho , J.‐G. Lee , J.‐M. Park , B.‐S. Lee , Y. Lee , C. Ko , Lab Chip 2007, 7, 565. 17476374 10.1039/b616115d

[18] D. S. Juang , T. D. Juang , D. M. Dudley , C. M. Newman , M. A. Accola , W. M. Rehrauer , T. C. Friedrich , D. H. O'Connor , D. J. Beebe , Nat. Commun. 2021, 12, 4317. 34262053 10.1038/s41467-021-24463-4 PMC8280165

[19] H. T. Ngo , M. Jin , A. Y. Trick , F.‐E. Chen , L. Chen , K. Hsieh , T.‐H. Wang , Anal. Chem. 2023, 95, 1159. 36562405 10.1021/acs.analchem.2c03897 PMC11250783

[20] S. M. Berry , E. T. Alarid , D. J. Beebe , Lab Chip 2011, 11, 1747. 21423999 10.1039/c1lc00004g PMC3244820

[21] J. S. Gootenberg , O. O. Abudayyeh , J. W. Lee , P. Essletzbichler , A. J. Dy , J. Joung , V. Verdine , N. Donghia , N. M. Daringer , C. A. Freije , C. Myhrvold , R. P. Bhattacharyya , J. Livny , A. Regev , E. V. Koonin , D. T. Hung , P. C. Sabeti , J. J. Collins , F. Zhang , Science 2017, 356, 438. 28408723 10.1126/science.aam9321 PMC5526198

[22] R. Aman , A. Mahas , M. Mahfouz , ACS Synth. Biol. 2020, 9, 1226. 32159950 10.1021/acssynbio.9b00507

[23] J. S. Chen , E. Ma , L. B. Harrington , M. Da Costa , X. Tian , J. M. Palefsky , J. A. Doudna , Science 2018, 360, 436. 29449511 10.1126/science.aar6245 PMC6628903

[24] Z. Huang , C. J. Lyon , T. Y. Hu , Nat. Rev. Bioengineer. 2023, 1, 230. 10.1038/s44222-023-00026-8 PMC9875755 37064656

[25] X. Ding , K. Yin , Z. Li , R. V. Lalla , E. Ballesteros , M. M. Sfeir , C. Liu , Nat. Commun. 2020, 11, 1. 32948757 10.1038/s41467-020-18575-6 PMC7501862

[26] A. Y. Trick , H. T. Ngo , A. H. Nambiar , M. M. Morakis , F.‐E. Chen , L. Chen , K. Hsieh , T.‐H. Wang , Lab Chip 2022, 22, 945. 35088790 10.1039/d1lc00820j PMC9035341

[27] Z. Chen , H. Zhu , J. AIDS Clin. Res. 2016, 7, 540. 26925300 10.4172/2155-6113.1000540 PMC4768831

[28] S. Berensmeier , Appl. Microbiol. Biotechnol. 2006, 73, 495. 17063328 10.1007/s00253-006-0675-0 PMC7080036

[29] H. He , R. Li , Y. Chen , P. Pan , W. Tong , X. Dong , Y. Chen , D. Yu , Sci. Rep. 2017, 7, 45199. 28332631 10.1038/srep45199 PMC5362898

[30] Z. Li , Y. Bai , M. You , J. Hu , C. Yao , L. Cao , F. Xu , Biosens. Bioelectron. 2021, 177, 112952. 33453463 10.1016/j.bios.2020.112952 PMC7774487

[31] A. A. Yazdi , A. Popma , W. Wong , T. Nguyen , Y. Pan , J. Xu , Microfluid Nanofluidics 2016, 20, 50.

[32] Z. Li , X. Ding , K. Yin , L. Avery , E. Ballesteros , C. Liu , Biosens. Bioelectron. 2022, 199, 113865. 34906838 10.1016/j.bios.2021.113865 PMC8653405

[33] N. Bhattacharjee , A. Urrios , S. Kang , A. Folch , Lab Chip 2016, 16, 1720. 27101171 10.1039/c6lc00163g PMC4862901

[34] N. L. Welch , M. Zhu , C. Hua , J. Weller , M. E. Mirhashemi , T. G. Nguyen , S. Mantena , M. R. Bauer , B. M. Shaw , C. M. Ackerman , S. G. Thakku , M. W. Tse , J. Kehe , M.‐M. Uwera , J. S. Eversley , D. A. Bielwaski , G. McGrath , J. Braidt , J. Johnson , F. Cerrato , G. K. Moreno , L. A. Krasilnikova , B. A. Petros , G. L. Gionet , E. King , R. C. Huard , S. K. Jalbert , M. L. Cleary , N. A. Fitzgerald , S. B. Gabriel , et al., Nat. Med. 2022, 28, 1083. 35130561 10.1038/s41591-022-01734-1 PMC9117129

[35] S. Klein , T. G. Müller , D. Khalid , V. Sonntag‐Buck , A.‐M. Heuser , B. Glass , M. Meurer , I. Morales , A. Schillak , A. Freistaedter , I. Ambiel , S. L. Winter , L. Zimmermann , T. Naumoska , F. Bubeck , D. Kirrmaier , S. Ullrich , I. Barreto Miranda , S. Anders , D. Grimm , P. Schnitzler , M. Knop , H.‐G. Kräusslich , V. L. Dao Thi , K. Börner , P. Chlanda , Viruses 2020, 12, 863. 32784757 10.3390/v12080863 PMC7472728

[36] S. L. Mitchell , K. S. George , J. Clin. Virology 2020, 128, 104429. 32425657 10.1016/j.jcv.2020.104429 PMC7227587

[37] J. S. Park , K. Hsieh , L. Chen , A. Kaushik , A. Y. Trick , T.‐H. Wang , Adv. Sci. 2021, 8, 2003564. 10.1002/advs.202003564 PMC7927608 33717855

[38] D. Liu , H. Shen , Y. Zhang , D. Shen , M. Zhu , Y. Song , Z. Zhu , C. Yang , Lab Chip 2021, 21, 2019. 34008614 10.1039/d0lc01222j

[39] H. Xiong , X. Ye , Y. Li , L. Wang , J. Zhang , X. Fang , J. Kong , Anal Chem. 2020, 92, 14297. 33073982 10.1021/acs.analchem.0c03364

[40] F. Tian , C. Liu , J. Deng , Z. Han , L. Zhang , Q. Chen , J. Sun , Sci. China Chem. 2020, 63, 1498. 32837510 10.1007/s11426-020-9800-6 PMC7387882

[41] X. Xie , T. Gjorgjieva , Z. Attieh , M. M. Dieng , M. Arnoux , M. Khair , Y. Moussa , F. Al Jallaf , N. Rahiman , C. A. Jackson , L. El Messery , K. Pamplona , Z. Victoria , M. Zafar , R. Ali , F. Piano , K. C. Gunsalus , Y. Idaghdour , Processes 2020, 8, 1.

[42] J. Sakai , N. Tarumoto , Y. Orihara , R. Kawamura , M. Kodana , N. Matsuzaki , R. Matsumura , K. Ogane , T. Kawamura , S. Takeuchi , K. Imai , T. Murakami , S. Maesaki , T. Maeda , J. Hospital Infection 2020, 105, 615. 10.1016/j.jhin.2020.05.025 PMC7242201 32446722

[43] H. Lin , W. Yu , K. A. Sabet , M. Bogumil , Y. Zhao , J. Hambalek , S. Lin , S. Chandrasekaran , O. Garner , D. Di Carlo , S. Emaminejad , Nature 2022, 611, 570. 36352231 10.1038/s41586-022-05408-3 PMC9645323

[44] J. Song , C. Liu , M. G. Mauk , J. Peng , T. Schoenfeld , H. H. Bau , Anal Chem. 2018, 90, 1209. 29226671 10.1021/acs.analchem.7b03834 PMC6310013

[45] Q. Bao , J. Sun , X. Fu , L. Sheng , Y. Ye , J. Ji , Y. Zhang , J. Wang , J. Ping , X. Sun , Small 2023, 19, 2207343. 10.1002/smll.202207343 37058127

