# Supplementary material for: Palm‐Sized Lab‐In‐A‐Magnetofluidic Tube Platform for Rapid and Sensitive Virus Detection
Source: Adv Sci (Weinh). 2024 Apr 17;11(23):2310066. doi: 10.1002/advs.202310066 (PMC11187901; doi:10.1002/advs.202310066)
Supplement: Supplementary file 1 — Supporting Information [file ADVS-11-2310066-s001.pdf]

ADVANCED SCIENCE
Open Access

Supporting Information

for *Adv. Sci.*, DOI 10.1002/advs.202310066

Palm-Sized Lab-In-A-Magnetofluidic Tube Platform for Rapid and Sensitive Virus Detection

*Ziyue Li, Shuo Zhang, Jiongyu Zhang, Lori Avery, David Banach, Hui Zhao and Changchun Liu**

Supporting Information

Palm-sized Lab-in-a-Magnetofluidic Tube Platform for Rapid and Sensitive Virus Detection

Ziyue Li[1,2], Shuo Zhang[1,2], Jiongyu Zhang[1,2], Lori Avery[3], David Banach [4], Hui Zhao [5], and Changchun Liu[1]*

1. *Department of Biomedical Engineering, University of Connecticut Health Center, Farmington, Connecticut 06030, United States*
2. *Department of Biomedical Engineering, University of Connecticut, Storrs, Connecticut 06269, United States*
3. *Department of Pathology and Laboratory Medicine, University of Connecticut Health Center, Farmington, Connecticut 06030, United States*
4. *Department of Medicine, Division of Infectious Diseases, University of Connecticut Health Center, Farmington, Connecticut 06030, United States*
5. *Department of Mechanical Engineering, University of Nevada, Las Vegas, Las Vegas, Nevada 89154, United States*

* **Corresponding author**

Dr. Changchun Liu

Department of Biomedical Engineering

University of Connecticut Health Center

263 Farmington Avenue

Farmington, CT 06030

Phone: (860)-679-2565

E-mail: chaliu@uchc.edu

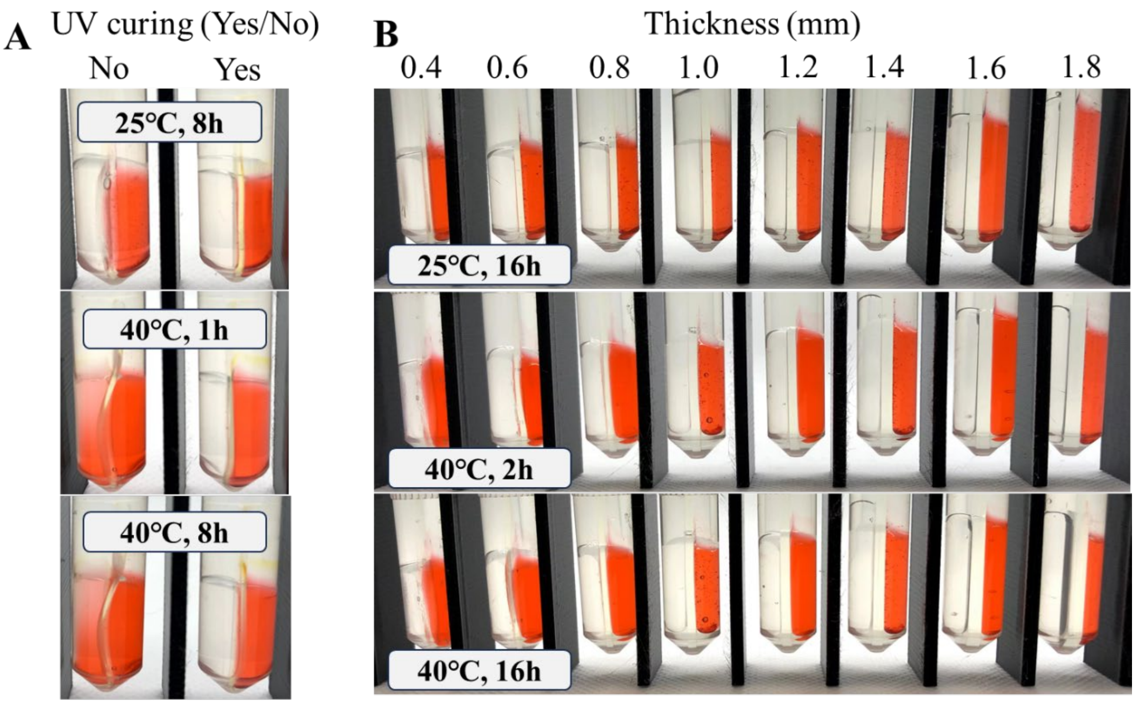

Figure S1. *Experimental optimization of 3D printed separators in the LIAMT device. A) Comparison of the 3D printed separator with and without UV curing. The LIAMT device was tested and compared at different incubation temperature and time (25℃ 8h; 40℃ 1h, 40℃ 8h). The UV curing process could significantly improve the mechanical strengthen of the 3D printed separator. B) The effect of the wall thickness of the separator on the liquid seal. We designed and tested the separators with different wall thickness (0.4 - 1.8 mm) at various incubation temperature and time (25℃ 16h; 40℃ 2h; 40℃ 16h). The 0.8 mm-thick separator is enough to completely prevent liquid leakage, enabling a seamless seal.*

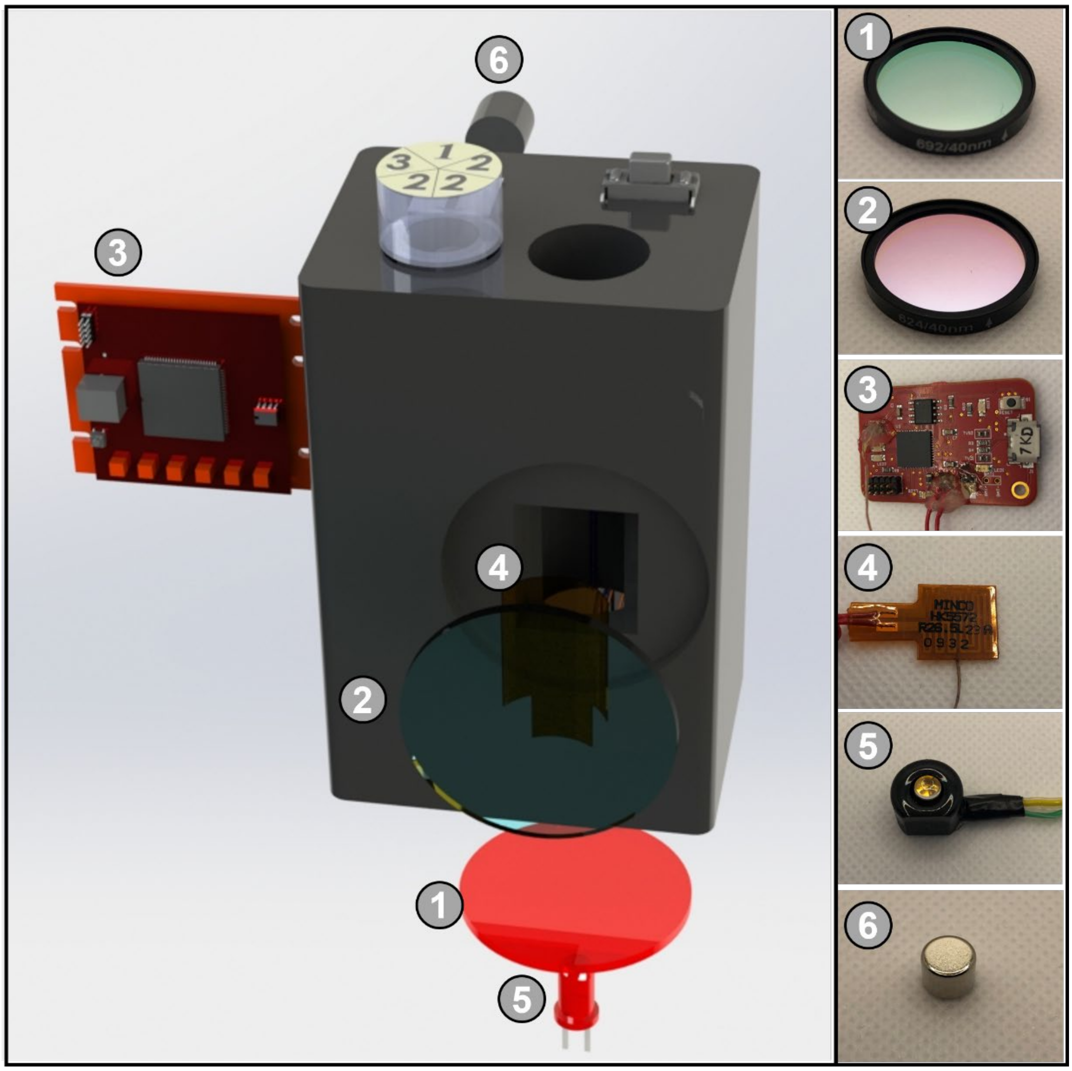

Figure S2. *Exploded view of the palm-sized processor, which includes: ① an excitation filter, ② an emission filter, ③ a control circuit board, ④ a flexible heater combined with a thermocouple wire, ⑤ a LED light, and ⑥ a neodymium magnet.*

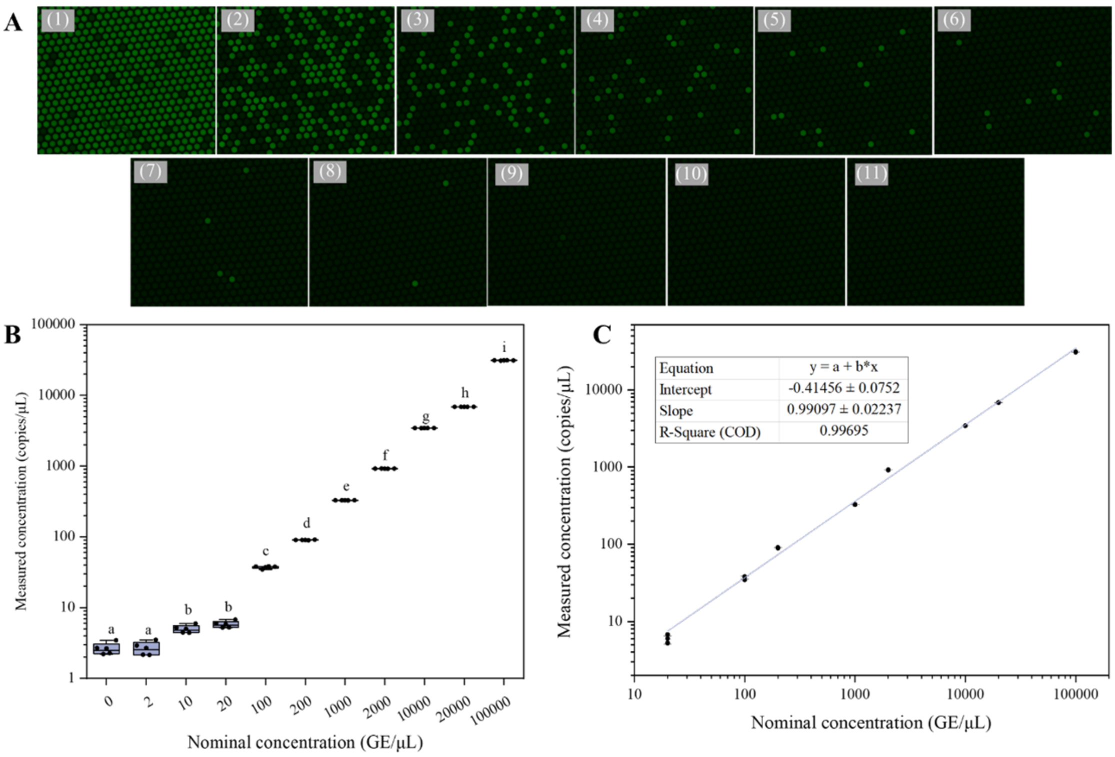

Figure S3. *Digital RT-PCR quantification of SARS-CoV-2 by QuantStudio 3D digital PCR chips. A) Fluorescence images of QuantStudio 3D digital PCR chips for detection of various SARS-CoV-2 concentrations. B) Absolute quantification of SARS-CoV-2 viral load by digital PCR in a dilution series. C) Linear regression of SARS-CoV-2 copy numbers and genome equivalents (GE) by digital PCR. Statistical significance was determined by performing an analysis of variance (ANOVA) test. Different letters were considered to be significantly different.*

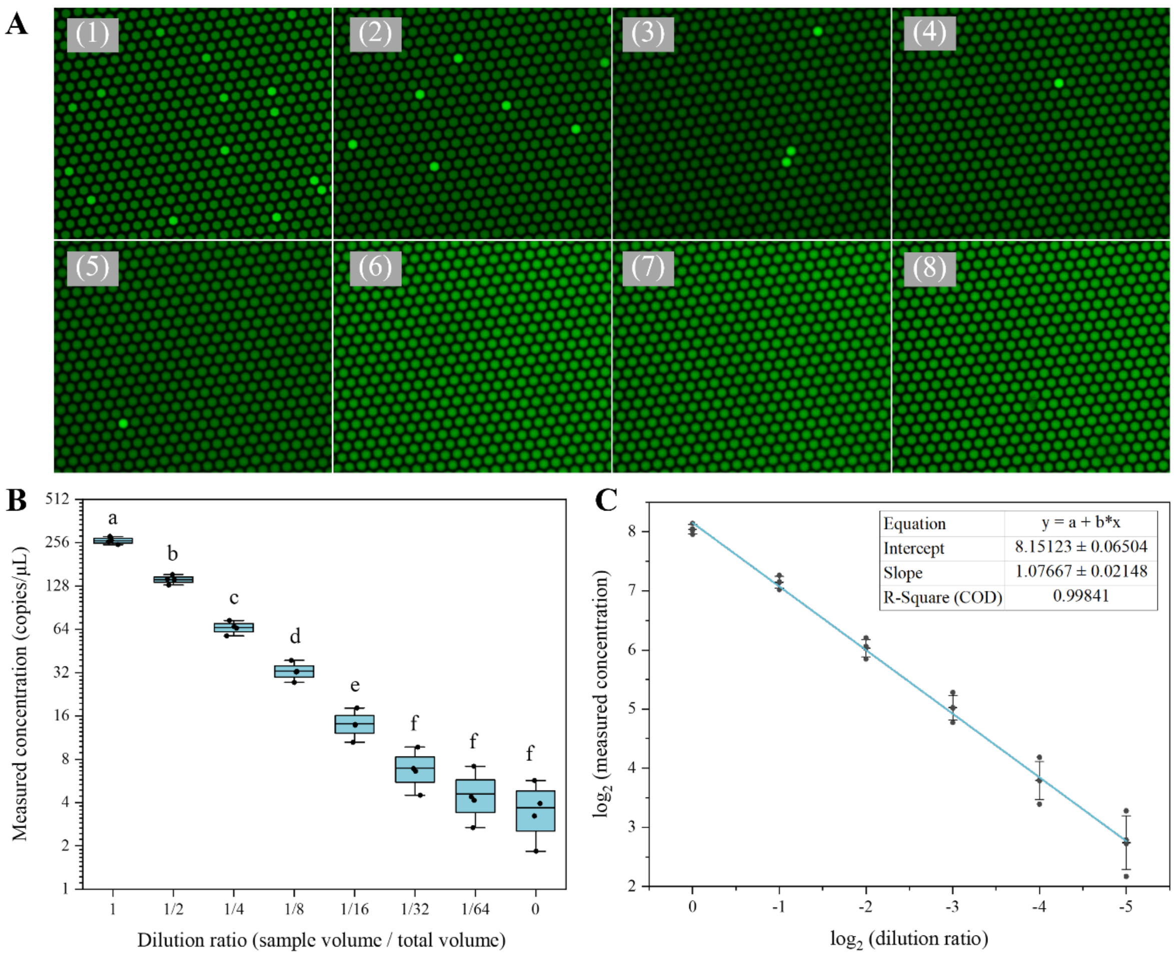

Figure S4. *Digital RT-PCR quantification of HIV by QuantStudio 3D digital PCR chips. A) Fluorescence images of QuantStudio 3D digital PCR chips for detection of various HIV serial dilutions. B) Absolute quantification of SARS-CoV-2 viral load by digital PCR in HIV serial dilutions. C) Linear regression of HIV copy numbers with serial dilutions by digital PCR. Statistical significance was determined by performing an ANOVA test. Different letters were considered to be significantly different.*

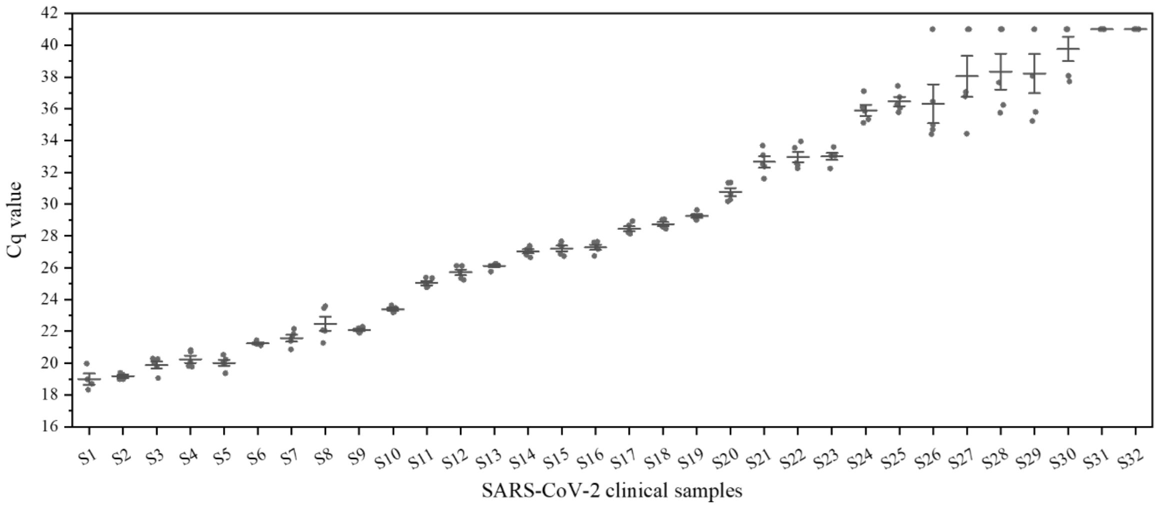

Figure S5. *Cq values of SARS-CoV-2 detection in 32 clinical samples by real-time RT-PCR.*

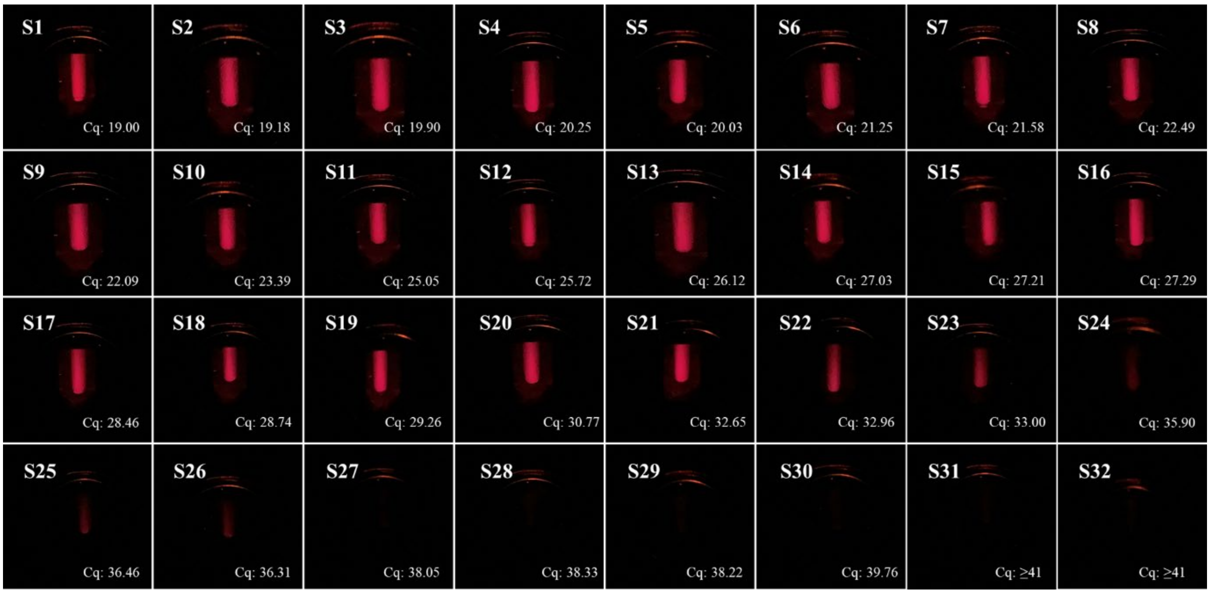

Figure S6. *Endpoint fluorescence images of the LIAMT device for the detection of different concentrations of SARS-CoV-2 in the spiked samples.*

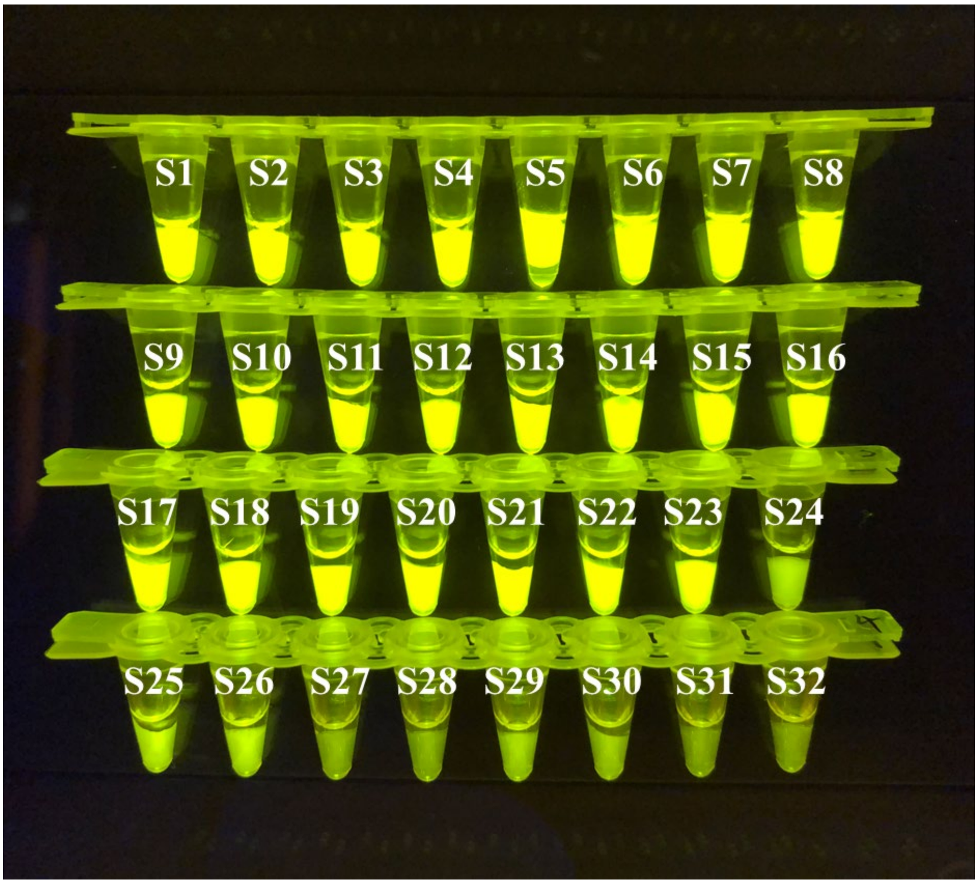

Figure S7. *Endpoint fluorescence detection of CRISPR reactions after separate RT-RPA preamplification for SARS-CoV-2 detection in 32 clinical samples.*

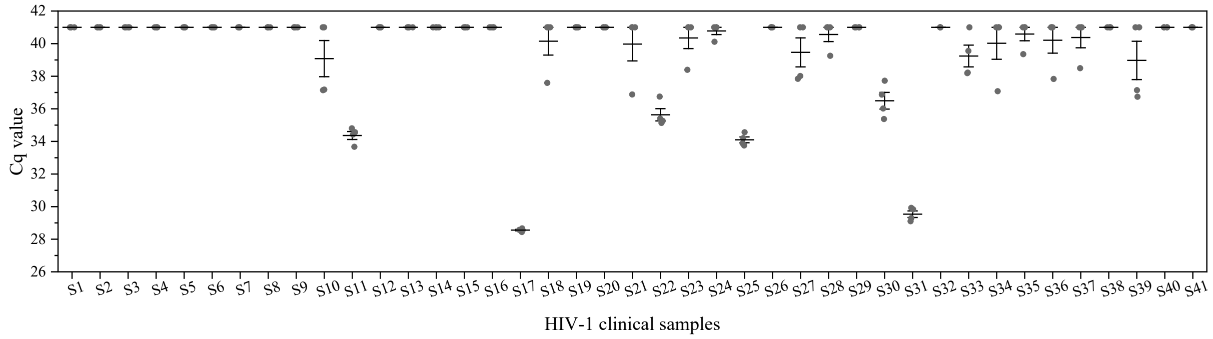

Figure S8. *Cq values of HIV detection in 41 clinical samples by real-time RT-PCR.*

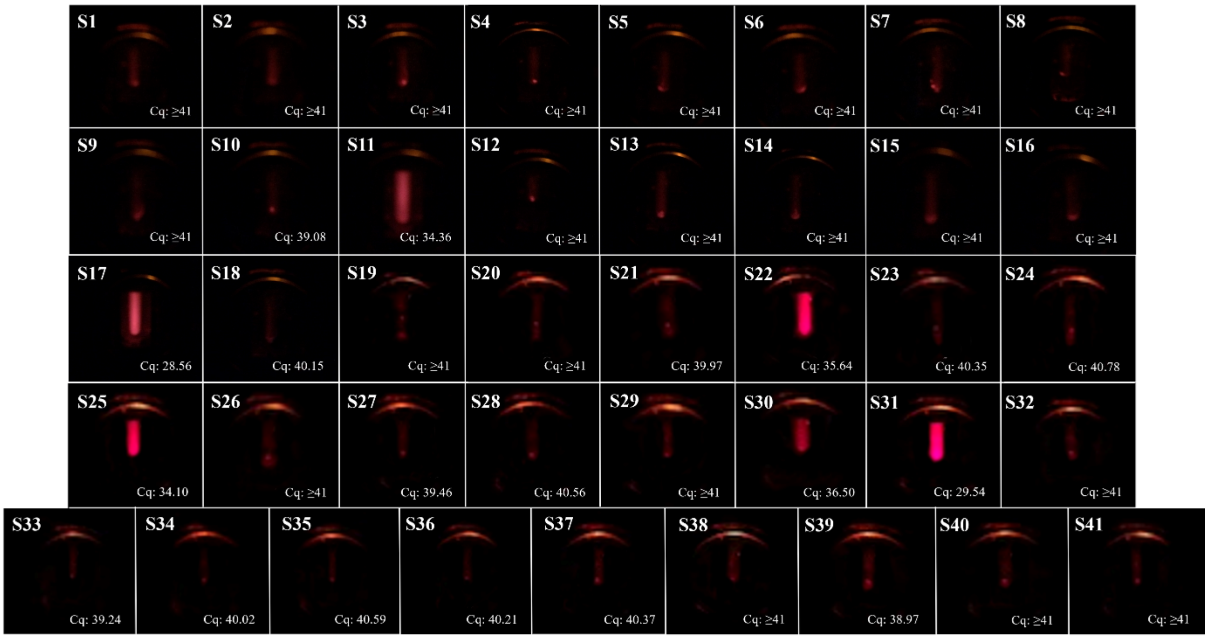

Figure S9. *Endpoint fluorescence images of the LIAMT device for the detection of different concentrations of SARS-CoV-2 in the spiked samples.*

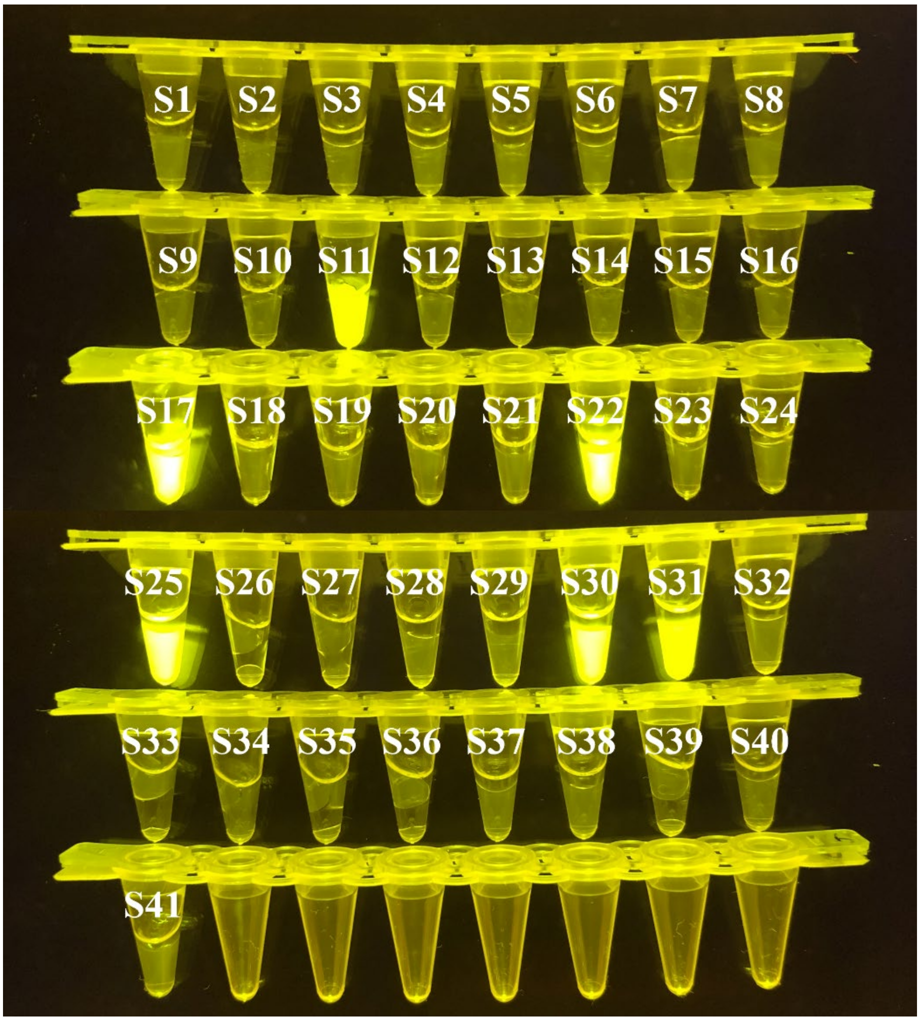

Figure S10. *Endpoint fluorescence detection of CRISPR reactions after separate RT-RPA preamplification for HIV detection in 41 clinical samples.*

Table S1. *Summary of different microfluidic molecular diagnostic technologies for pathogen detection.*

Name	Target*	Pre-amplification	CRISPR tech	Microfluidic tech	Readout	Reaction Time (min)	LOD	Portable Detection	Contaimination Safety	Clinical Sample	Sample-to-Result solution	Reference
deCOViD	SARS-CoV-2	RT-RPA	LbCas12a	Silicon chip	Flurescence	15-30	1 copies/μL	No	No	Nasopharyngeal swab	No	[1]
mCARMEN	respiratory virus panel (21 viruses)	RT-PCR	Cas12a, Cas13a	Integrated fluidic circuits	Flurescence	130-160	10 copies/μL	No	No	Nasopharyngeal swab	No	[2]
MI-IF-RPA	SARS-CoV-2	RT-RPA	No	Chip	Lateral flow	30	1 copies/μL	Yes	Yes	Nasopharyngeal swab	No	[3]
--	human coronaviruses	RT-LAMP	No	Centrifugal chip	Flurescence	40	10 copies/μL	Yes	Yes	--	No	[4]
--	SARS-CoV-2	RT-LAMP	No	Centrifugal chip	Flurescence	70	2 copies/reaction	No	Yes	Oropharyngeal swab (spiked)	Yes	[5]
--	SARS-CoV-2	RT-PCR	No	Chip	Flurescence	--	1 copies/μL	No	No	Nasopharyngeal swab	No	[6]
GeneSoC	SARS-CoV-2	RT-PCR	No	Chip	Flurescence	15	10 copies/reaction	Yes	Yes	Nasopharyngeal swab	No	[7]
LIAMT	SARS-CoV-2, HIV	RT-RPA	Cas12a	Tube	Flurescence	60	73.4, 63.9 copies/μL	Yes	Yes	Nasopharyngeal swab, Plasma sample	Yes	This work

* SARS-CoV-2, Severe acute respiratory syndrome coronavirus 2; HIV-1, Human immunodeficiency virus type 1.

-- No data.

Table S2. *List of all oligonucleotide sequences used for SARS-CoV-2 detection.*

Oligonucleotide	Sequence
**crRNA**	
LbCas12a-crRNA-SARS-CoV-2	UAAUUUCUACUAAGUGUAGAU**UUGAACUGUUGCGACUACGU**
**Target**	
SARS-CoV-2 RNA Control 2	*from* Twist Bioscience Part#102024 (GenBank-MN908947.3)
**RPA Primers**	
FP_SARS-CoV-2	CGGCAGTCAAGCCTCTTCTCGTTCCTCATC
RP_SARS-CoV-2	CAGACATTTTGCTCTCAAGCTGGTTCAATC
**Reporter**	
ssDNA-Cy5Q	/5Cy5/TTATTTTATT/3BHQ_2/
ssDNA-FQ	/56-FAM/TTATT/3IABkFQ/
**PCR**	
SARS-CoV-2 Forward Primer	*from* IDT Part#10006821
SARS-CoV-2 Reverse Primer	*from* IDT Part#10006822
SARS-CoV-2 PCR Probe	*from* IDT Part#10006823

Table S3. *List of all oligonucleotide sequences used for HIV detection* [8].

Oligonucleotide	Sequence
**crRNA**	
Lba Cas12a-crRNA1-HIV-1	UAAUUUCUACUAAGUGUAGAU**AUCCCAUUCUGCAGCUUCCUCAUU**
Lba Cas12a-crRNA2-HIV-1	UAAUUUCUACUAAGUGUAGAU**UUGCACCAGGCCAGAUAAGA**
**Target**	
HIV-1 plasmid	CCAGAAGTAATACCCATGTTTTCAGCATTATCAGAAGGAGCCACCCCACAAGATTTAAACACCATGCTAAACACAGTGGGGGGACATCAAGCAGCCATGCAAATGTTAAAAGAAACCATCAATGAGGAAGCTGCAGAATGGGATGAAATGCATCCCGTGCAGGCAGGGTTTGTTGCACCAGGCCAGATAAGAGATCCAAGGGGAAGTGACATAGCAGGAACTACCAGTACCCTTCAGGAACAAATAGGATGGATGACAAGTAATCCACCTATCCCAGTAGGAGAAATCTATAAAAGATGG
AcroMetrix HIV-1 high controls	*from* ThermoFisher Scientific Cat.#964003
ACCURUN 315 series 400 HIV-1 RNA positive control	*from* SeraCare Life Sciences Cat.#2020-0092
**RPA Primers**	
FP_HIV-1	CAAGCAGCCATGCAAATGTTAAAAGAAACCATC
RP_HIV-1	GTAGTTCCTGCTATGTCACTTCCCCTTGGATC
**dsDNA**	
RPA amplicons	CAAGCAGCCATGCAAATGTTAAAAGAAACCATCAATGAGGAAGCTGCAGAATGGGATGAAATGCATCCCGTGCAGGCAGGGTTTGTTGCACCAGGCCAGATAAGAGATCCAAGGGGAAGTGACATAGCAGGAACTAC
**Reporter**	
MB-Invertase reporter	MB/streptavidin/BsI probe
BsI probe	/5Biotin/AAAAAAAAAAAATCAATCTCGGATTCCGGAGACAAGTTGAAGAGAACCTGGGGGAGTGCGAGTTCACAGATGAGTTGACAGATGAGTAAAAAAAAAAAA/3Invertase
ssDNA-FQ	/56-FAM/TTATT/3IABkFQ/
**PCR**	
HIV-1 Forward Primer	CAAGCAGCCATGCAAATGTTA
HIV-1 Reverse Primer	GGTAGTTCCTGCTATGTCACTTC
HIV-1 Probe	/56-FAM/TTGGATCTC/ZEN/TTATCTGGCCTGGTGC/3IABkFQ/

References

[1] J. S. Park, K. Hsieh, L. Chen, A. Kaushik, A. Y. Trick, T.-H. Wang, *Advanced Science* **2021**, *8*, 2003564.

[2] N. L. Welch, M. Zhu, C. Hua, J. Weller, M. E. Mirhashemi, T. G. Nguyen, S. Mantena, M. R. Bauer, B. M. Shaw, C. M. Ackerman, S. G. Thakku, M. W. Tse, J. Kehe, M.-M. Uwera, J. S. Eversley, D. A. Bielwaski, G. McGrath, J. Braidt, J. Johnson, F. Cerrato, G. K. Moreno, L. A. Krasilnikova, B. A. Petros, G. L. Gionet, E. King, R. C. Huard, S. K. Jalbert, M. L. Cleary, N. A. Fitzgerald, S. B. Gabriel, G. R. Gallagher, S. C. Smole, L. C. Madoff, C. M. Brown, M. W. Keller, M. M. Wilson, M. K. Kirby, J. R. Barnes, D. J. Park, K. J. Siddle, C. T. Happi, D. T. Hung, M. Springer, B. L. MacInnis, J. E. Lemieux, E. Rosenberg, J. A. Branda, P. C. Blainey, P. C. Sabeti, C. Myhrvold, *Nat Med* **2022**, *28*, 1083.

[3] D. Liu, H. Shen, Y. Zhang, D. Shen, M. Zhu, Y. Song, Z. Zhu, C. Yang, *Lab Chip* **2021**, *21*, 2019.

[4] H. Xiong, X. Ye, Y. Li, L. Wang, J. Zhang, X. Fang, J. Kong, *Anal Chem* **2020,** *92*, 14297.

[5] F. Tian, C. Liu, J. Deng, Z. Han, L. Zhang, Q. Chen, J. Sun, *Sci China Chem* **2020**, *63*, 1498.

[6] X. Xie, T. Gjorgjieva, Z. Attieh, M. M. Dieng, M. Arnoux, M. Khair, Y. Moussa, F. Al Jallaf, N. Rahiman, C. A. Jackson, L. El Messery, K. Pamplona, Z. Victoria, M. Zafar, R. Ali, F. Piano, K. C. Gunsalus, Y. Idaghdour, *Processes* **2020**, *8*, 1.

[7] J. Sakai, N. Tarumoto, Y. Orihara, R. Kawamura, M. Kodana, N. Matsuzaki, R. Matsumura, K. Ogane, T. Kawamura, S. Takeuchi, K. Imai, T. Murakami, S. Maesaki, T. Maeda, *Journal of Hospital Infection* **2020**, *105*, 615.

[8] Z. Li, N. Uno, X. Ding, L. Avery, D. Banach, C. Liu, *ACS nano*. **2023** 17, 3966-75.
